# Incidence, Outcome, and Risk Factors of Cardiovascular Surgery-Associated Disseminated Intravascular Coagulation: A Single-Center Retrospective Study

**DOI:** 10.3390/jcm11133633

**Published:** 2022-06-23

**Authors:** Norihisa Yasuda, Koji Goto, Yoshihide Kuribayashi, Yoshifumi Ohchi, Takaaki Kitano

**Affiliations:** 1 Department of Anesthesiology and Intensive Care, Faculty of Medicine, Oita University, 1-1 Idaigaoka, Hasamamachi, Yufu City 879-5593, Japan; kuribayashi@oita-u.ac.jp (Y.K.); ohchi-yo@oita-u.ac.jp (Y.O.); tkitano@oita-u.ac.jp (T.K.); 2 Department of Anesthesiology, Oita Urological Hospital, 2-1-32 Nagahamamachi, Oita City 870-0023, Japan; gotoita510@gmail.com

**Keywords:** cardiovascular surgery, disseminated intravascular coagulation (DIC), DIC scoring system, pre-operative DIC score

## Abstract

Cardiovascular surgery is highly invasive, with a risk of postoperative coagulopathy due to various factors such as bleeding. Coagulopathy can progress to disseminated intravascular coagulation (DIC), which complicates various clinical conditions. However, no study to date has reported on DIC associated with cardiovascular surgery. Therefore, we investigated retrospectively the incidence, outcome, and risk factors of cardiovascular surgery-associated DIC in our institute. All patients who underwent cardiovascular surgery and were admitted to our intensive care unit between January 2016 and December 2017 were included in this study. The Japanese Association for Acute Medicine (JAAM) DIC score was calculated using our institute’s database at the following time points: preoperatively, postoperative day 1 (POD1), POD3, and POD7. Data regarding surgery, 90-day mortality, and risk factors of DIC were also collected and analyzed by multiple regression. In total, 553 patients were considered eligible for analysis. Median age of eligible patients was 72 years, with a 90-day mortality rate of 1.4%. Patients with DIC at POD7 had higher Sequential Organ Failure Assessment (SOFA) score, preoperative JAAM DIC scores, and a longer anesthesia time than those without DIC. Female sex, preoperative DIC score, and anesthesia time were found to be risk factors for DIC.

## 1. Introduction

Disseminated intravascular coagulation (DIC) complicates a variety of clinical conditions. Sepsis and malignant tumors are major underlying diseases, and pregnancy and acute pancreatitis, as well as surgery and trauma, can also lead to DIC. Gando et al., reported that trauma, burn, and surgery contributed to the greatest number of DIC cases diagnosed according to the Japanese Association for Acute Medicine (JAAM) DIC scoring system [1,2]. Cardiovascular surgery is highly invasive and often accompanied by severe bleeding, resulting in prolonged coagulopathy and subsequent development of DIC. Moreover, surgery with cardiopulmonary bypass (CPB) requires the use of anticoagulants to prevent hemostasis during CPB. Thrombocytopenia and dilutional coagulopathy may progress during the postoperative period, which can lead to DIC and prolonged ICU stays. The dilutional coagulopathy can particularly cause reduced fibrinogen levels, and hypofibrinogenemia contributes to various pathological events. Simurda, T. et al., reviewed that hypofibrinogenemia can occur in 50% of patients underwent complex cardiac surgery [3,4]. However, no study to date has reported on cardiovascular surgery-associated DIC. Therefore, the present study aimed to investigate the incidence, mortality, and risk factors of cardiovascular surgery-associated DIC in patients admitted to our institute.

## 2. Materials and Methods

### 2.1. Study Design

Using our institute’s database of patients admitted to the intensive care unit (ICU) between January 2016 and December 2017, we selected patients who underwent cardiovascular surgery according to the following exclusion criteria: age < 18 years, severe liver dysfunction, use of mechanical circulatory support (e.g., intra-aortic balloon pumping (IABP), venous-arterial extracorporeal membrane oxygenation (V-A ECMO), left ventricular assist device (LVAD)), and history of re-thoracotomy due to bleeding or cardiac tamponade.

We collected data including platelet counts, prothrombin time-international normalized ratio (PT-INR), fibrinogen/fibrin degradation products (FDP), and systemic inflammatory response syndrome (SIRS) scores using our database, and calculated JAAM DIC scores at the following time points: preoperatively, postoperative day 1 (POD1), POD3, and POD7 (Table 1). Patients with JAAM DIC scores ≥ 4 were diagnosed with DIC, and data were compared between those with JAAM DIC scores ≥ 4 (DIC group) and those with JAAM DIC scores < 4 (no-DIC group). To assess the severity of disease, Acute Physiology and Chronic Health Evaluation-II (APACHE-II) and Sequential Organ Failure Assessment (SOFA) scores were calculated at POD0. The following operative data were also collected: operation time, anesthesia time, CPB time, and intraoperative blood loss. Data on continuous renal replacement therapy (CRRT) and 90-day mortality were also obtained.

### 2.2. Statistical Analysis

Data were presented as median and interquartile ratio (IQR) or number as appropriate. To compare between the DIC and no-DIC groups, the Mann–Whitney test or Fisher exact test was used. A log-rank test was used to analyze 90-day mortality. In addition, multiple logistic regression analysis was performed to assess risk factors for cardiovascular surgery-associated DIC. *p* < 0.05 was considered statistically significant. All statistical analyses were carried out with Statflex Statistical Software version 7.0 (Artech Co., Ltd., Osaka, Japan).

## 3. Results

### 3.1. Patient Characteristics

**Figure 1:**
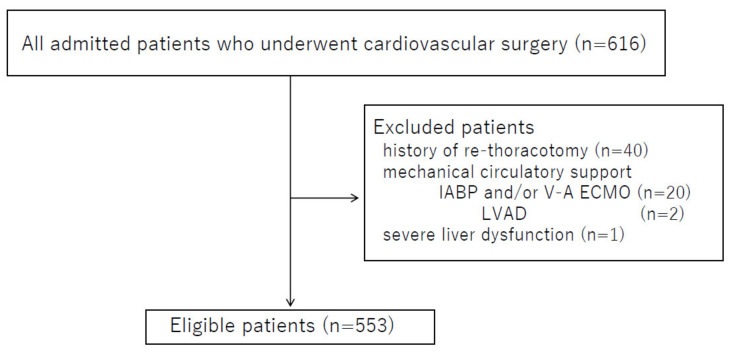
Schematic of patient recruitment. IABP: intra-aortic balloon pumping, V-A ECMO: venous-arterial extracorporeal membrane oxygenation, LVAD: Left ventricular assist device.

A total of 553 patients were included in this study (Figure 1). Patient characteristics are shown in Table 2. Median age was 72 years, and median APACHE-II and SOFA scores at POD0 were 14 points and 7 points, respectively. Forty-four patients met the DIC criterion (i.e., JAAM DIC scores ≥ 4) preoperatively, with a median preoperative JAAM DIC score of 0 points. The numbers of patients who underwent surgery with CPB, mechanical ventilation, and CRRT were 299 (54.1%), 393 (71.1%), and 74 (13.4%), respectively. Median ICU length of stay was 4 days, with a 90-day mortality rate of 1.4% (7/553 patients). Types of surgery are shown in Table 3; valve surgery was highest in number. Median operation and anesthesia times were 310 min and 449 min, respectively. Median blood loss during surgery was 580 mL.

### 3.2. Incidence of DIC

The numbers of patients with DIC at POD1 and POD3 were 228 and 141, respectively. However, since patients with DIC at POD1 and POD3 had higher intraoperative blood loss than those without DIC, we decided not to diagnose DIC at POD1 and POD3 given the possible influence of bleeding on DIC scores. At POD7, 127 patients still remained in the ICU, and intraoperative blood loss did not differ significantly between the DIC (*n* = 66) and no-DIC (*n* = 61) groups (Table 4). Therefore, we diagnosed these 66 patients with cardiovascular surgery-associated DIC (11.9%). In our analysis, the incidence of DIC did not differ significantly between the types of surgery. Compared to the no-DIC group, the DIC group had significantly higher SOFA and pre-operative DIC scores and a longer anesthesia time (Table 5). However, there was no significant difference in 90-day mortality. To assess risk factors for cardiovascular surgery-associated DIC, multiple logistic regression analysis was performed with age, sex, APACHE-II score, SOFA score, preoperative DIC score, anesthesia time, use of CPB, and blood loss as independent variables. Finally, female sex, preoperative DIC score, and anesthesia time were significant risk factors for DIC (Table 6). The regression analysis suggested that use of CPB did not affect the incidence of DIC.

## 4. Discussion

In this study, we investigated the incidence, outcome, and risk factors of cardiovascular surgery-associated DIC in patients admitted to our institute. At POD7, 66 patients were diagnosed with DIC, which accounted for roughly 11.9% of all patients included in this study. Patients with DIC had a significantly higher SOFA score, longer anesthesia time, and longer ICU length of stay than those without DIC. However, the incidence of DIC did not affect 90-day mortality. Female sex, pre-operative DIC score, and anesthesia time were found to be risk factors for DIC.

DIC results from tissue factor-mediated initiation of systemic coagulation activation, decreased physiologic anticoagulant activity, and impaired endogenous fibrinolysis. A variety of underlying diseases, such as infections and malignant tumors, can lead to DIC, which may also contribute to mortality [5]. Trauma and surgery can also cause DIC. In particular, tissue injury and shock during cardiovascular surgery, as well as the use of CPB and anticoagulation, can damage the coagulation system. All patients who undergo cardiovascular surgery will have a coagulation profile of DIC for some time after surgery, but most of them will recover from hemostatic disorder immediately in 3–5 days after surgery. However, we experienced prolonged coagulopathy and DIC occurred in some patients after surgery, and no study has reported on the incidence of cardiovascular surgery-associated DIC. To the best of our knowledge, this is the first study to retrospectively examine the incidence, mortality, and risk factors of cardiovascular surgery-associated DIC.

With regard to the mechanism of post-trauma coagulopathy, Spahn et al., reported that inflammation, loss of coagulation factors, and activation of fibrinolysis are related to the progression of coagulopathy [6]. Kornblith et al., found tissue injury to be a cause of protein C activation, impaired thrombin formation, and dysregulated fibrinolysis [7]. The mechanism of trauma-induced coagulopathy may be partly applicable to surgery-induced coagulopathy and DIC. In the case of surgery with CPB, an association between CPB and postoperative coagulopathy has been suggested [8].

While trauma-induced coagulopathy occurs in the early stage of trauma injury, probably within a few days, our patients with DIC were diagnosed at 7 days postoperatively (POD7). Therefore, the status of coagulation and fibrinolysis likely differs between the two conditions (trauma vs. surgery). The trauma-induced coagulopathy is driven by hemorrhagic shock and extensive tissue disruption. On the other hand, cardiovascular surgery-induced coagulopathy is mainly driven by surgical tissue injury and CPB-related coagulopathy. These differences partly explain the results.

In the present study, DIC scores of ≥4 at POD1 and POD3 were significantly associated with intraoperative blood loss. However, DIC scores of ≥4 at POD7 were not, suggesting that some patients might have had prolonged coagulopathy that took a longer time to recover. We thought that postoperative infection or sepsis is a reason for the delayed recovery from coagulopathy. However, in our institute, all patients who underwent cardiovascular surgery were administered antibiotics postoperatively and serum procalcitonin levels were routinely measured in all patients. The patients with DIC in our study did not have clinical manifestations of infection. It remains unclear what factors contribute to prolonged coagulopathy and DIC.

Zhang et al., conducted a study on chronic DIC and aneurysm and reported that the proportion of females was significantly higher in the DIC group than in the no-DIC group, although the reason for this was unclear [9]. Estrogen may play a role in thrombosis formation, as some studies have reported that estrogen increases the risk of both arterial and venous thrombosis [10]. However, female patients enrolled in this study were older and possibly had decreased hormone secretion. Therefore, the reason females are a risk factor for DIC still remains unclear.

We found that patients with a higher preoperative DIC score are more likely to develop DIC at POD7. This means that preoperative patients with coagulopathy may still suffer from coagulopathy one week after surgery, with subsequent progression to DIC. These findings suggest the need to closely monitor the time course of coagulation and fibrinolysis parameters in preoperative patients with coagulopathy.

While anesthesia time was also a significant risk factor for DIC, its impact on DIC may be limited given the very small odds ratio (1.016). Operation time did not have a significant impact on DIC. A large-scale study will be needed to further investigate the impact of anesthesia time on DIC.

This study used the JAAM DIC scoring system to diagnose DIC, because it is a commonly used system in clinical settings in Japan. Gando et al., reported that the JAAM DIC scoring system is comparable to the ISTH DIC scoring system; the most frequently noted underlying diseases in their study were trauma, burn, and surgery [1,2]. Demma et al., reported that the JAAM DIC scoring system is more useful than the ISTH DIC scoring system in predicting mortality in patients with DIC after surgery with CPB [11]. Meanwhile, Grafender et al., reported that ISTH DIC scores predict outcomes in non-septic patients admitted to a cardiovascular ICU [12]. Based on these reports, we considered the JAAM DIC scoring system to be a well-validated tool for evaluating cardiovascular surgery-associated DIC.

In our institute, anticoagulants are often used to treat DIC. Some patients in the present study were administered recombinant human thrombomodulin and antithrombin concentrates, which are usually used to treat septic DIC in clinical settings and have been reported to be effective [13,14]. In a previous case report, recombinant thrombomodulin was useful for treating chronic DIC in a patient with dissecting aortic aneurysm [15]. In the present study, the proportion of patients with anticoagulation therapy was too small to evaluate the efficacy of anticoagulants. Further investigation will be necessary to examine the effectiveness of anticoagulants in the treatment of cardiovascular surgery-associated DIC.

This study has many limitations. First, since this study was a retrospective study, various confounders might have affected the results. Second, we could not follow patients after they were discharged from the ICU. Thus, it is unknown whether any of our patients developed DIC after ICU discharge. Third, we examined intraoperative blood loss but not blood loss after ICU admission, which could have affected the incidence of DIC (although we excluded patients who underwent re-thoracotomy). Fourth, we did not examine the volume of transfusion during ICU stay. Some patients supplemented cryoprecipitate due to the decreased fibrinogen levels. This may also have influenced the results. Fifth, we did not collect the data regarding anticoagulation therapy and antiplatelet therapy before surgery, which may have had an impact on the results, though the impact would have been minimal considering the half-time of those drugs. Sixth, our results suggest that the use of CPB did not influence the incidence of DIC. However, because of the small sample size, we still cannot exclude the possibility that use of CPB affects postoperative coagulopathy and DIC. Future prospective, multi-center studies are warranted to further evaluate coagulopathy and DIC after cardiovascular surgery.

## 5. Conclusions

We investigated the incidence, outcome, and risk factors of cardiovascular surgery-associated DIC in our institute. The incidence of DIC was approximately 11.6%, and the presence of DIC had no impact on 90-day mortality. Female sex, preoperative DIC score, and anesthesia time were found to be risk factors for DIC.

## Figures and Tables

**Table 1:** JAAM DIC Scoring system.

Systemic Inflammatory Response Syndrome Criteria	Score
≥3	1
0–2	0
Platelet counts (×10^4^/μL)	
<8.0, or a ≥50% decrease within 24 h	3
≥8.0 and <12.0, or a ≥30% decrease within 24 h	1
≥12.0	0
PT-INR	
≥1.2	1
<1.2	0
FDP (μg/mL)	
≥25	3
≥10 and <25	1
<10	0
DIC diagnosis	
≥4 points	

JAAM: Japanese Association for Acute Medicine, DIC: disseminated intravascular coagulation, PT-INR: prothrombin time-international normalized ratio, FDP: fibrin/fibrinogen degradation products.

**Table 2:** Baseline characteristics.

Variables	*n* = 553
Age (year)	72 (64–79)
Sex (male/female), *n*	342/211
APACHE-II score at POD0	14 (10–17)
SOFA score at POD0	7 (4–9)
Preoperative JAAM DIC score	0 (0–1)
Mechanical ventilation (y/n)	393/160
ICU length of stay (days)	4 (3–6)
CRRT (y/n)	74/479
90-day mortality, *n*	7

Values are presented as median (25th/75th percentile). *n*: number, APACHE-II: Acute Physiology and Chronic Health Evaluation-II, SOFA: Sequential Organ Failure Assessment, JAAM DIC: Japanese Association for Acute Medicine-Disseminated Intravascular Coagulation, CRRT: continuous renal replacement therapy.

**Table 3:** Type of operation and other operative data.

Variables		*n* = 553
Type of operation	Valve surgery	178 (32%)
	Thoracic aortic surgery	128 (23%)
	CABG surgery	91 (17%)
	Abdominal aortic surgery	105 (19%)
	Stent-graft surgery	32 (6%)
	Other procedures	19 (3%)
Operation time (min)		310 (239–401)
Anesthesia time (min)		449 (357–543)
Use of CPB (*n*)		299
CPB time (min)		148 (72–210)
Intraoperative blood loss (mL)		580 (300–1120)

Values are presented as median (25th/75th percentile) or number (*n*). CABG: Coronary artery bypass graft, CPB: cardio-pulmonary bypass.

**Table 4:** Incidence of cardiovascular surgery-associated DIC.

	Total (*n*)	DIC Group (*n*)	No-DIC Group (*n*)	Comparison of Intraoperative Blood Loss between the Two Groups
POD1	536	228	308	*p* = 0.0058
POD3	360	141	219	*p* = 0.0013
POD7	127	66	61	*p* = 0.19 (n.s)

*n*: number, DIC: disseminated intravascular coagulation, POD: postoperative day, n.s: not significant.

**Table 5:** Cardiovascular surgery-associated DIC at POD7.

Variables	DIC Group (*n* = 66)	No-DIC Group (*n* = 61)	*p* Value
Age (years)	76 (65–80)	74 (63–80)	0.348
Sex (M/F)	36/30	41/20	0.144
APACHE-II at POD0	17 (14–22)	16 (12–19)	0.337
SOFA at POD0	11 (8–12)	9 (6–12)	0.038
Preoperative JAAM DIC score	2 (1–4)	1 (0–3)	0.026
Anesthesia time (min)	488 (413–650)	446 (347–525)	0.011
Intraoperative blood loss (mL)	803 (470–1803)	670 (320–1230)	0.191
Use of CPB (*n*)	21	14	0.096
90-day mortality (*n*)	3	1	0.369

Values are presented as median (25th/75th percentile) or number (*n*). DIC: disseminated intravascular coagulation, APACHE-II: Acute Physiology and Chronic Health Evaluation-II, SOFA: Organ Failure Assessment, JAAM DIC: Japanese Association for Acute Medicine-DIC, CPB: cardiopulmonary bypass.

**Table 6:** Multiple logistic regression analysis on the incidence of DIC.

Variables	Coefficient	Odds Ratio	95% CI	*p* Value
Females	1.133	3.106	1.291–7.472	0.0113
Preoperative JAAM DIC score	0.297	1.323	1.060–1.651	0.0134
Anesthesia time	0.003	1.016	1.001–1.050	0.0165

CI indicates confidence interval. DIC: disseminated intravascular coagulation, JAAM DIC: Japanese Association for Acute Medicine-DIC.

## Data Availability

The data regarding the findings of this study are available from the corresponding author upon reasonable request.
